# Chemical Composition and Food Potential of *Pachymerus nucleorum* Larvae Parasitizing *Acrocomia aculeata* Kernels

**DOI:** 10.1371/journal.pone.0152125

**Published:** 2016-03-31

**Authors:** Ariana Vieira Alves, Eliana Janet Sanjinez Argandoña, Adelita Maria Linzmeier, Claudia Andrea Lima Cardoso, Maria Lígia Rodrigues Macedo

**Affiliations:** 1 Faculty of Exact Sciences and Technology, Federal University of Grande Dourados, Dourados, Mato Grosso do Sul, Brazil; 2 Federal University of Fronteira Sul, Realeza, Paraná, Brazil; 3 Course of Chemistry, State University of Mato Grosso do Sul, Dourados, Mato Grosso do Sul, Brazil; 4 Laboratory of Protein Purification and Biological Functions, Departament of Natural Science, Federal University of Mato Grosso do Sul, Campo Grande, Mato Grosso do Sul, Brazil; USDA-ARS, UNITED STATES

## Abstract

Insect consumption as food is culturally practiced in various regions of the world. In Brazil, there are more than 130 species of edible insects registered, from nine orders, among which stands out the Coleoptera. The larva of the beetle *Pachymerus nucleorum* Fabricius, 1792, grows into the bocaiuva fruit (*Acrocomia aculeata* (Jacq.) Lodd. Ex Mart., 1845), which has proven nutritional quality. The aim of this work was to evaluate the nutritional potential of *P*. *nucleorum* larvae compared to bocaiuva kernels for human consumption. Proteins were the second largest portion of the larvae nutritional composition (33.13%), with percentage higher than the bocaiuva kernels (14.21%). The larval lipid content (37.87%) was also high, very close to the kernels (44.96%). The fraction corresponding to fatty acids in the oil extracted from the larvae was 40.17% for the saturated and 46.52% for the unsaturated. The antioxidant activity value was 24.3 uM trolox/g of oil extracted from larvae. The larvae tryptic activity was 0.032±0.006 nmol BAPNA/min. Both the larvae and the bocaiuva kernel presented absence of anti-nutritional factors. These results favor the use of *P*. *nucleorum* larvae as food, which are a great protein and lipid sources with considerable concentrations of unsaturated fatty acids compared to the bocaiuva kernel.

## Introduction

The Food and Agriculture Organization of the United Nations (FAO) estimates that in the period 2010 to 2012 approximately 870 million people were undernourished, indicating that 12.5% of world population, one in eight human beings are affected by malnutrition [1].

Despite the substantial increase in food production in the last fifty years [2], estimates indicate a population increase reaching nine billion people by 2050, which will result in reduced food availability, especially animal protein [3,4]. In 2013, after the International Conference on Forests for Food Security and Nutrition, FAO published a report [5] that encourages insect consumption as a way to fight hunger and promote food security, since the insects are protein sources.

Insect consumption as food is culturally practiced in various regions of the world except in developed regions like Europe and North America. Jongema [6] points out an estimated 2000 species of edible insects, consumed by more than 3.000 ethnic groups [7].

Brazil has an extremely rich and exuberant biosociodiversity, presenting one of the greatest biological diversity of the planet and huge cultural diversity. Costa Neto and Ramos-Elorduy et al. [8] registered in Brazil 135 species of edible insects, from nine orders, among which stands out the Coleoptera, with 22 species reported as human food. *Pachymerus nucleorum* Fabricius, 1792 (Chrysomelidae, Bruchinae) is a common beetle in palm coconuts, such as babassu (*Attalea speciosa* Mart. Ex Spreng.), piassava (*Attalea funifera* Mart. Ex Speg.), licuri (*Syagrus coronata* (Mart.) Becc.) and bocaiuva (*Acrocomia aculeata* (Jacq.) Lodd. ex Mart., 1845), whose larvae develops inside the coconut feeding exclusively of the kernel [8].

Among the palm trees found in the State of Mato Grosso do Sul, there is the bocaiuva. Studies have shown that the fruit (pulp and kernel) is rich in carotenoids and fatty acids, having high nutritional [9, 10, 11] and anti-inflammatory [12, 13] capability.

Fatty acids are classified as saturated and unsaturated; the unsaturated have important functions in the human body, for example, in maintaining the immune system in inflammatory processes. The fatty acid levels found in bocaiuva oil suggest an effective action in these pathologies [13].

In this context, this work aimed to evaluate the nutritional potential of *P*. *nucleorum* larvae compared to bocaiuva kernel, expecting that the insect had equal or superior nutritional characteristics of the kernel.

## Material and Methods

### Material

The bocaiuva (*Acrocomia aculeata*, Arecaceae) fruits were collected in Dourados, MS, Brazil at Estadual University of Mato Grosso do Sul campus with the permission of the institution. The field studies did not involve endangered or protected species. They’re taken to the laboratory and opened to remove the kernels and those wich were infested by *Pachymerus nucleorum* the larvae were removed. Thereafter, the kernels were dried in an oven with air circulation at 40°C for 72 hours, crushed and stored in a cool environment. These fruits are deposited in the Herbarium DDMS of the Federal University of Grande Dourados—UFGD under the number No. 4783, PEREIRA, Z. V.

The larvae were washed, packed in expanded polystyrene boxes and frozen at -6°C, keeping stored at this temperature until analysis time. The *Pachymerus nucleorum* identification was carried out by Dra. Cibele Stramare Ribeiro-Costa from the Federal University of Parana (UFPR), the Brazilian expert in the group.

### Chemical analysis

The analysis were carried out on nutritional composition, fatty acid composition, antioxidant activity, tryptic activity and anti-nutritional factors of bocaiuva kernels and *Pachymerus nucleorum* larvae.

### Nutritional composition

The parameters assessed were: moisture content in oven [14]; fixed mineral residue (ashes) by burning the material in an oven at 550°C [14]; lipids by petroleum ether extraction in a Soxhlet equipment [14]; protein content quantity by determining the nitrogen present in the sample through the Kejldahl method, using the conversion factor of 6.25 [14]; and fibers by acid and alkaline extraction [15].

Carbohydrates were determined by difference (100g sample—g of moisture-ashes-lipid-protein-fiber). The energy value was calculated using the Atwater coefficient, which establishes 4 kcal/g of sample for protein and carbohydrates and 9 kcal/g of sample for lipids [16].

### Fatty acid composition

The kernels and larvae lipids were extracted following the Bligh and Dyer [17] method. The triglycerides transesterification were carried out by transferring approximately 50 mg of the lipid matters extracted to 15 ml falcon tubes, and add to them 2 ml of n-heptane. The mixture was stirred until fatty matter complete dissolution, then 2 ml of KOH2 mol/l in methanol was add. The mixture was again stirred for about 5 minutes and after the separation of phases, 1 ml of the upper phase (heptane and methyl esters of fatty acids) was transferred to 1.5 ml Eppendorf vials. The vials were hermetically sealed, protected from light and stored in a freezer at -18°C for further chromatographic analysis.

The fatty acid composition was determined by gas chromatography using a gas chromatograph with flame ionization detector (GC-FID). The elution was carried out using a fused silica capillary column with 100 m x 0.25 mm x 0.20 μm (SP-2560). The oven temperature was programmed to begin at 100°C, maintained for 1 min and then raised to 170°C at 6.5°C/min.

Subsequently, another increase from 170 to 215°C was performed at 2.75°C/min, maintaining this temperature for 12 min. Finally, a last increase was performed from 215 to 230°C at 40°C/min. The injector and detector temperatures were 270 and 280°C, respectively.

The 0.5 μl samples were injected in "split" (1:20) mode, using nitrogen as carrier gas with flow rate of 1 ml/min. The identification of methyl esters of fatty acids was performed by comparison with the sample compounds retention times with the standards (Sigma) eluted under the same conditions of the samples.

### Analysis of antioxidant activity

The extract was prepared from the mixture of 1 g of oil and 50 ml of hydromethanol (50%) solution. After resting for 60 min, the material was centrifuged (4000 rpm) for 15 min and the supernatant was removed. To make the second extraction, 40 ml of acetone (70%) was added to the sediment, following the procedures from the first extraction. The supernatants from the two extractions were mixed, transferred to a flask (100 ml) and the volume completed with distilled water to form the extract.

The radical ABTS^•+^ (2, 2-Azino BIS 3-ethylbenzo thiazoline 6 sulfonic acid diammoninum) was formed by reaction of ABTS^•+^ (7 mM) with potassium persulfate (140 mM), the mixture reacted for 16 h at room temperature in absence of light, forming the radical solution. The radical solution was diluted with ethanol until absorbance of 0.70 (± 0.05) at 734 nm (spectrophotometer Biospectro) to perform the analysis. Aliquots of 30 μl of sample were added to 3 ml of the ABTS^•+^diluted solution and the mixture absorbance was registered after 6 min. The antioxidant activity was calculated using the standard curve of 6-hydroxy-2,5,7,8-tetrametilchroman-2-carboxylic acid (Trolox). The standard curve was prepared from Troloxethanolic solutions at concentrations of 100; 500; 1000; 1500 and 2000 μM [18]. The results were expressed as μM of Trolox/g of extract. Each determination was performed in triplicate.

### Analysis of tryptic and chymotryptic activities

The tryptic and chymotryptic activities were carried out in microplates [19]. The assay utilizes the hydrolysis of chromogenic substrates N-α Benzoyl-D-L-Arginine p-nitroanilide (BApNA) to trypsin and Succynil Alanine Alanine PF p-nitroanilide (SAAPFPNA) for chymotrypsin.

Larval tryptic activity was performed by incubating the samples with Tris-HCl 50 mM, pH 8.0 to a final volume of 70 μl. After adding substrate, the assay time was 30 min at 37°C. The results of this analysis were expressed as nmol/BApNA/min and UI/mL. Larval chymotryptic activity was analyzed by incubating the samples with Tris-HCl 50 mM, pH 8.0 to a final volume of 100 μl. After the substrate addition, the test time was 10 minutes at 37°C and the reaction was read in a Multiskan Go Microplate Reader at 410 nm. The results of this analysis were expressed as nmol/SAAPFPNA/min and UI/ml.

The enzymatic assays for analysis of larvae anti-tryptic and anti-chymotryptic potential, were carried out adding 10 μl of bovine trypsin for the anti-tryptic and 10 μl bovine chymotrypsin for the anti-chymotryptic, in order to determine whether they have inhibitory action on these enzymes. After the addition of Tris-HCl 50 mM, pH 8.0, the respective substrates were added as described in the larvae tryptic and chymotryptic assays, continuing the incubation and reading at 410 nm. Each assay and sample had three replicates. Reactions were read in a Multiskan Go Microplate Reader at 410 nm.

### Statistical analysis

All analyzes were performed in triplicate and results were expressed as mean and standard deviation. Comparisons of mean values between groups were performed by analysis of variance (ANOVA) and the differences compared by Tukey test at p <0.05 significance level, using the software Statistica 8.0 [20] and Prism 3.0 [21].

## Results and Discussion

### Nutritional composition

The nutritional composition results for *P*. *nucleorum* larvae and bocaiuva kernels were compared to beef and soybean nutritional composition values (Table 1), established by the Brazilian Table of Food Composition [22].

**Table 1:** Nutritional composition of *Pachymerus nucleorum* (Coleoptera, Chrysomelidae) larvae, *Acrocomia aculeata* (Arecaceae) kernels and conventional foods.

						Carbohydrate		
Samples	Moisture* (g/100g)	Moisture (g/100g)	Ashes (g/100g)	Lipid (g/100g)	Protein (g/100g)	Fiber (g/100g)	Starch (g/100g)	Energy value (kcal/100g)
*P*. *nucleorum* larvae	35.15±0.64^a^	54.22^a^	3.15±0.24^a^	37.87±0.97^a^	33.13±0.98^a^	15.37±1.24^a^	-	473
*A*. *aculeate* kernels	5.13±0.20^b^	5.54^b^	2.23± 0.10^a^	44.96±0.79^b^	14.21±0.85^b^	39.17±1.18^b^	-	461
Beef **	52.70	-	1.90	67.23	35.31	N.A.	-	358
Soybean**	5.80	-	5.00	14.60	36.00	-	38.40	404

Values presented on a dry basis. Results expressed with ± standard deviation, n = 3.

* Moisture on a wet basis.

** Brazilian Table of Food Composition (TACO), 2011. NA = not applicable. Means with different superscript letters in the same column differ significantly (p <0.05).

The *P*. *nucleorum* larvae showed 35.15% moisture (Table 1), below the beef (52.7%). The bocaiuva kernel and soybean had similar moisture content (5.13% and 5.80% respectively). The other constituents were calculated on a dry basis to avoid interference of moisture content in the samples.

The ashes contents in the larvae (3.15%) and kernels (2.23%) were similar and higher than the beef (1.9%) (Table 1). The ashes value found meets the daily mineral intake recommendation, which is approximately 3 g [23]. Therefore, 30g of *P*. *nucleorum* larvae meets 31% of the daily minerals requirement in humans.

The lipid content of larvae (37.87%) was lower than the kernels (44.96%) and higher than the soybean (14.60%) (Table 1). From the energy point of view, lipids are important because they produce 9 kcal per gram of food when oxidized in the body [23]. In some countries, this energy source contributes with 30–40% of total energy consumed in food [23]. Lipids are structural components of all tissues and indispensable in cell membranes structure and cell organelles [24,25,26]. They also stimulate the body carotenoids absorption, providing bioavailability of these compounds [27].

The insect protein digestibility is comparable with conventional meat [28]. Proteins are the second largest portion of the *P*. *nucleorum* larvae nutritional composition (Table 1). The larvae protein content (33.13%) was similar to the value reported by Ramos-Elorduy *et al*. [29] for the same species (33.05%). This value was higher than the bocaiuva kernel (14.21%), and close to the beef (35.31%) and soybean (36.0%) (Table 1). These results shows the *P*. *nucleorum* larvae potential as protein supplement for people interested in increasing protein intake.

Animal protein is superior to plant; therefore, the best protein supplements should include some animal protein [30]. Many of these products contain milk-derived protein, whose production causes environmental impact much greater than the insects [30]. The insect based products has a relatively low acceptability barrier, since they aim consumers with nutritional and environmental awareness, and the protein source is not visible or taste distinguishable (ex.: replacing soybean powder by insects powder does not alter the appearance, taste or texture of the product) [30]. Thus, the insects may be a high quality protein ingredient for high standard protein supplement in the food industry.

The Ordinance No.27/1998 of the Health Surveillance National Agency [31] states that a food can be considered rich in fiber when presenting more than 6% fiber content. The *P*. *nucleorum* larvae and bocaiuva kernels are in this category, since they presented respectively 15.37% and 39.17% of fibers. Rich fiber food consumption is associated with reduced risk of cardiovascular disease, lower blood glucose and lipid levels associated with decreased hyperinsulinemia. Moreover, high consumption implies lower risk for obesity development [32].

Regarding the energy, *P*. *nucleorum* larvae can be (473 kcal) compared to the kernel (461 kcal) and stood out compared to beef (358 kcal) and soybeans (404 kcal), with higher value (Table 1). The consumption of 100 g of larvae would represent approximately one third of the daily energy amount needed [23].

### Fatty acid composition and antioxidant activity

The main fatty acid found in the oil extracted from *P*. *nucleorum* larvae was the oleic acid (44.09%), followed by the acids lauric (33.87%), stearic (3.91%) and linoleic (3.96%) (Table 2). The main fatty acid found in the oil extracted from the bocaiuva kernels was the lauric (39.56%), oleic (33.04%), myristic (7.99%), palmitic (6.87%), and linoleic (3.03%).

**Table 2:** Composition of fatty acids in total lipids of oils extracted from *Pachymerus nucleorum* (Coleoptera, Chrysomelidae) larvae and *Acrocomia aculeata* (Arecaceae) kernels.

Fatty acids (%)	*P*. *nucleorum* larvae	*A*. *aculeata* kernels
**Caproic Acid** (C6:0)	-	0.33±0.01
**Caprylic acid** (C8:0)	-	2.89±0.03
**Lauric acid** (C12:0)	33.87±0.50^a^	39.56±0.70^b^
**Myristic acid** (C14:0)	2.45±0.01^a^	7.99±0.05^b^
**Palmitic acid** (C16:0)	2.01±0.01^a^	6,87±0.04^b^
**Stearic acid** (C18:0)	3.91±0.01^a^	3.14±0.02^a^
**Arachidonic acid** (C20:0)	0.02±0.01^a^	0,11±0.01^a^
**Palmitoleic acid** (C16:1)	0.05±0.01^a^	0.08±0.01^a^
**Oleic acid** (C18:1)	44.09±0.10^a^	33.04±0.08^b^
**Linoleic acid** (C18:2)	3,96±0,01^a^	3,03±0,01^a^
α**-Linolenic acid** (C18:3)	0,04±0,01^a^	0,09±0,01^a^
**Eicosapentaenoic Acid—CLA** (C20:5)	0.07±0.01	-
∑ AGS^A^	42.26	60.89
∑ AGM^B^	44.95	33.12
∑ AGPI^C^	4.00	3.12

^A^ AGS = saturated fatty acids;

^B^ AGM = monounsaturated fatty acids;

^C^ AGPI- polyunsaturated fatty acids. Results are expressed as ± standard deviation, n = 3. Means with different superscript letter in the same row differ significantly (p <0.05).

The fraction corresponding to saturated fatty acids in the oil extracted from larvae was 42.26% and 48.95% unsaturated (Table 2). The kernel oil had 60.89% of saturated fatty acids and 36.24% unsaturated. These results confirm the high content of unsaturated fatty acids in larva and kernel oils, due mainly to the high content of oleic acid.

Polyunsaturated fatty acids from the omega-3 series (C18:3 and C20:5) and omega-6 (C18:2) can act in preventing cardiovascular disease and cancer [24]. The high fatty acids concentration in the oil affects its antioxidant activity, which is highly desired in the human diet [24].

The conjugated linoleic acid (CLA), present in meat products, has been shown antioxidant activity [33,34]. The CLA may decrease the saturated fatty acids accumulation in cell membranes, making these membranes less susceptible to oxidation, with less potential of oxidative damage to the cellular components [33,34].

The antioxidant activity of *P*. *nucleorum* larvae oil, measured by the ABTS method, was 24.3 μM Trolox/g, higher than conventional oils such as soybean (2.2 μM Trolox/g) and sunflower (1.17 μM Trolox/g) [35].

There is evidence that high concentrations of monounsaturated fatty acids (AGM) are beneficial to the human body. The carbohydrates replacement by AGM increases the HDL cholesterol concentration and the saturated fatty acids replacement by AGM decreases the LDL cholesterol level in the blood [24,36,37].

### Tryptic activity and anti-nutritional factors

Protein digestion begins in the stomach, where they decompose into proteases, peptones and large polypeptides; and continues in the intestine, where they undergo most of digestion in the intestinal part known as duodenum. In this part, the proteins undergo the action of enzymes secreted by the pancreas, especially the proteolytic enzymes trypsin, chymotrypsin and caboxipoli-pepitdase [38]. Among the proteolytic enzymes, trypsin stands out because it is being found in digestive systems of many vertebrates that require hydrolyze and absorb proteins. Therefore, the trypsin activity is essential in the hydrolysis process and absorption of proteins, since these molecules are too large to be absorbed by the intestine [38].

The *P*. *nucleorum* larvae and bocaiuva kernels were analyzed for tryptic, anti-tryptic, chymotryptic, and anti-chymotryptic activities. The trypsin enzyme activity of larvae was 0.032±0.006 nmol BAPNA/min. The trypsin activity was not verified in the kernel sample. The presence of this enzyme suggests that this larvae consumption as food may lead to a higher availability of amino acids.

Strengthening this hypothesis, the absence of anti-nutritional factors in the larvae and kernels was verified by the anti-tryptic and anti-chymotryptic analysis. The presence of these factors may hinder the proteins absorption and utilization from the diet, because the trypsin inhibitor binds to the trypsin enzyme, responsible for proteins digestion [39].

## Conclusions

The results indicated that *Pachymerus nucleorum* larvae are great protein and lipid sources for humans with significant levels of unsaturated fatty acids compared to the bocaiuva kernel.

The *P*. *nucleorum* larvae and bocaiuva kernels presented absence of anti-nutritional factors, favoring their use as food for humans.

The rational exploitation of *Pachymerus nucleorum* as food for humans can be done by the food industry in protein supplement production for athletes and people who want to increase the protein consumption; and used as an incentive for the insect species conservation, their host plants and their natural habitats.
